# Therapeutic Applications of Metal and Metal-Oxide Nanoparticles: Dermato-Cosmetic Perspectives

**DOI:** 10.3389/fbioe.2021.724499

**Published:** 2021-08-20

**Authors:** Sharadwata Pan, Thomas B. Goudoulas, Jaison Jeevanandam, Kei Xian Tan, Shamik Chowdhury, Michael K. Danquah

**Affiliations:** ^1^ TUM School of Life Sciences, Technical University of Munich, Freising, Germany; ^2^ CQM-Centro de Química da Madeira, MMRG, Universidade da Madeira, Campus da Penteada, Funchal, Portugal; ^3^ School of Materials Science and Engineering, Nanyang Technological University, Nanyang, Singapore; ^4^ School of Environmental Science and Engineering, Indian Institute of Technology Kharagpur, Kharagpur, India; ^5^ Department of Chemical Engineering, University of Tennessee, Chattanooga, TN, United States

**Keywords:** therapeutic, metal, metal oxide, nanoparticle, dermal, rheology, cosmetics

## Abstract

Invention of novel nanomaterials guaranteeing enhanced biomedical performance in diagnostics and therapeutics, is a perpetual initiative. In this regard, the upsurge and widespread usage of nanoparticles is a ubiquitous phenomenon, focusing predominantly on the application of submicroscopic (< 100 nm) particles. While this is facilitated attributing to their wide range of benefits, a major challenge is to create and maintain a balance, by alleviating the associated toxicity levels. In this minireview, we collate and discuss particularly recent advancements in therapeutic applications of metal and metal oxide nanoparticles in skin and cosmetic applications. On the one hand, we outline the dermatological intrusions, including applications in wound healing. On the other hand, we keep track of the recent trends in the development of cosmeceuticals *via* nanoparticle engrossments. The dermato-cosmetic applications of metal and metal oxide nanoparticles encompass diverse aspects, including targeted, controlled drug release, and conferring ultraviolet and antimicrobial protections to the skin. Additionally, we deliberate on the critical aspects in comprehending the advantage of rheological assessments, while characterizing the nanoparticulate systems. As an illustration, we single out psoriasis, to capture and comment on the nanodermatology-based curative standpoints. Finally, we lay a broad outlook and examine the imminent prospects.

## Introduction

**FIGURE 1:**
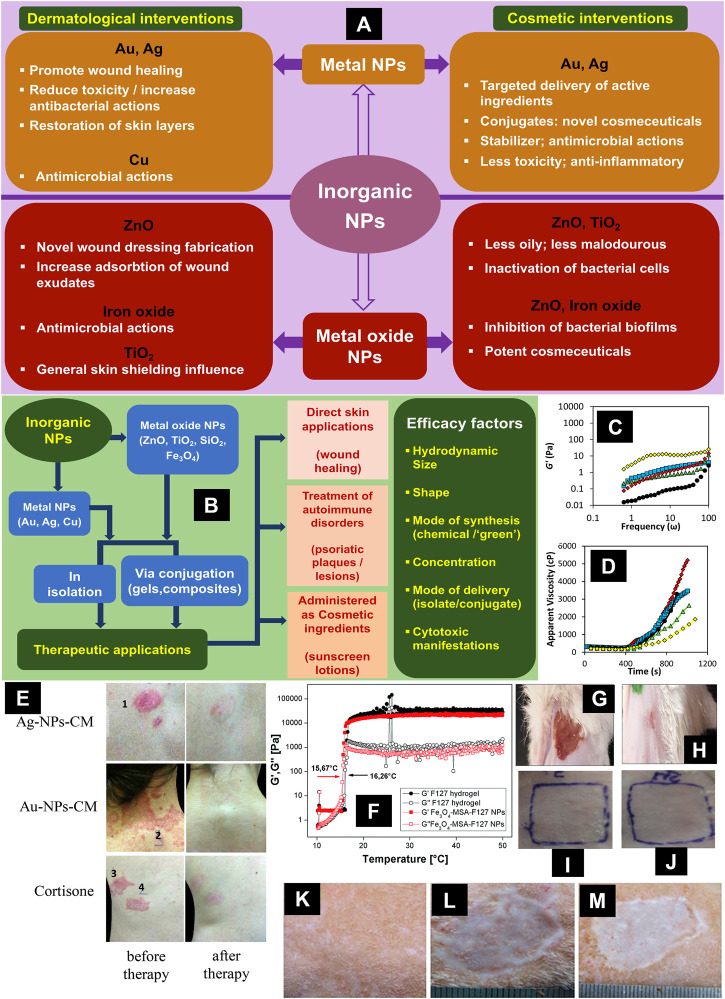
**(A)**: Representative schematic of various dermatological and cosmetic interventions of the metal and metal oxide NPs considered in the current minireview. **(B)**: Representative schematic illustrating an all-inclusive premise of the current minireview. **(C,D)**: Efficacy of rheological characterizations in assessing mechanical and gelation properties of polymer gels incorporated with different metal oxide Nps. Linear elastic modulus (G′) as a function of imposed angular oscillation frequency at different NP concentrations **(C)**. Temporal evolution of the apparent viscosity during gelation in the presence and absence of different NPs at different polymer concentrations **(D)**. Legend: Open diamond—Chromium (III) oxide; closed diamond—Aluminium oxide; closed triangle—Silicon dioxide; closed square—Magnesium oxide; closed circle—gel without NPs. Reproduced and adapted from Pérez-Robles et al. (2020). Open Access. © 2019 Pérez-Robles et al. **(E)**: Noteworthy reduction in psoriasis plaques, scaling and erythema in human volunteers, after 6 weeks of administration of gold and silver NPs, *via* the *Cornus mas* conjugates. Reproduced with permission from Crisan et al. (2018). © 2018 John Wiley and Sons A/S. **(F)**: Efficacy of rheology in assessing the temperature dependence of the mechanical properties (linear viscoelastic moduli: G′ and G″) of superparamagnetic iron oxide NPs dispersed in Pluronic F127 hydrogel. Reproduced from Gonçalves et al. (2017). Open Access. © The Royal Society of Chemistry 2017. **(G,H)**: Efficacy of gold NPs in wound healing. Burn wound position on mouse dorsal skin treated with F1 formula (AuNPs-PF127), mouse before application; day 1 **(G)**, mouse after end of treatment; day 9 **(H)**. Reproduced from Arafa et al. (2018). Open Access. © Arafa et al. (2018). **(I,J)**: Efficacy of metal oxide NPs in a dermato-cosmetic application. Response for conventional titanium dioxide cream after 48 h **(I)**. Response for titanium dioxide NPs cream after 48 h (**J**). Reproduced with permission from Singh and Nanda (2014). © 2014 Society of Cosmetic Scientists and the Société Française de Cosmétologie. **(K–M)**: Efficacy of silver NP loaded collagen/chitosan scaffolds (NAg-CCS) in wound healing. Gross views of normal skin **(K)** and wounds treated by NAg-CCS **(L)** and CCS **(M)** at day 60 post transplantation, respectively. Ultra-thin autografts were transplanted at day 10. Reproduced from You et al. (2017). Open Access. © You et al., 2017. Abbreviation: NP—nanoparticle, NPs—nanoparticles.

The widespread applications of nanomaterials in biomedical sciences embody one of the paramount advancements in research targeting the utilization of submicroscopic particles. Evidently, this transcends numerous diverse domains, such as the mode of synthesis, mechanisms of vesicular carrier formation, risks and toxicity assessment, diagnostic and healing potential, source or origin of raw materials, characteristic size and charge range, among others (Jeevanandam et al., 2017; Nnaji et al., 2018; Tan et al., 2018; Tan et al., 2019; Fytianos et al., 2020; Has and Pan, 2020; Tan et al., 2020; Pan et al., 2021a; Jeevanandam et al., 2021). To highlight the perspective, one may consider the recent developments in the enhanced utilization of gold-based nanomaterials in several capacities towards generating antimicrobial actions in general, and antibacterial effects, in particular (Mutalik et al., 2020; Yougbaré et al., 2021). In the current minireview, we will only focus on the therapeutic aspects of metal and metal oxide nanoparticles in dermato-comsetology, attributing to the fact that we recurrently tend to overlook that dermatology and cosmetology are often intertwined. This is again frequently, but not limited to, with respect to drug testing and performance, active ingredient permeation to skin strata, effects of components on the material properties, high end applications in wound healing and tissue engineering, and tuning of mechanical properties of novel nanocomposites with the established benchmarks (Pan et al., 2019a; Pan and Germann, 2019; Pan and Germann, 2020; Pan et al., 2021b; Malhotra et al., 2021). Much of these nanomaterial benefits cater to their unique properties, including trivial size, robustness of the colloidal moiety, greater surface area, higher bioavailability, among others (Niska et al., 2018; Salatin et al., 2019; Naskar and Kim, 2020). While the basic intention during employment of nanoparticles in both cosmetic and skin applications (such as wound healing) is identical, similar problems and their sources complicate their scaling up and commercialization. For instance, although novel cosmeceuticals developed via integration of metal and metal oxide nanoparticles is abundant at lab scale, integration of a robust quality system (Pan et al., 2013; Ramanunny et al., 2021) to optimize the production process is yet to be established. Similarly, rampant issues with associated toxicity levels present colossal challenges to the large-scale employment and optimization of inorganic nanomaterials in dermatological complications (Jeevanandam et al., 2019). Hopefully, circumstances are gradually transforming, with substantial efforts focusing more on “greener” or natural methods of fabrication, as well as high throughput characterizations, for early detection and evasion of irregularities. This minireview focuses on the particularly recent advancements in this regard, and offers a concise bundle of the current state-of-the-art. The overall theme of the current minireview is illustrated schematically in Figure 1B.

## Dermatological Interventions

### Metal Nanoparticles

With respect to skin applications, metal nanoparticles have mainly been utilized in countering dermatological infections caused by adventitious agents, as well as to facilitate wound healing. Arafa et al. (2018) recently reported on the development of heat receptive gels containing gold nanoparticles, with noteworthy antibacterial characteristics (against *Staphylococcus aureus*) and heightened lesion remedial abilities. Particularly the capabilities of both silver and gold nanoparticles to advance wound healing and fight antimicrobial infections, are aptly highlighted in very recent articles [(Mihai et al., 2019; Naskar and Kim, 2020; Ramanunny et al., 2021); and references therein], catering to features such as ability to regulate cytokine secretion, inducing differentiation and proliferation of specific kinds of cells (keratinocytes, myofibroblasts), hindering bacterial reproduction, circumventing on-site microbial settlement, exhibiting both “bactericidal” and “bacteriostatic” properties, among others. Naskar and Kim (2020) have also reported that silver nanoparticles embedded in unique scaffolds (guar gum, polyvinyl alcohol, polyethylene fabric, etc.) promote wound healing via rapid cessation of lesions, alleviated toxicity and rapid restoration of the on-site skin stratum. While certain advantages are intrinsic to the employment of such inorganic nanoparticles, either in pure form or as composites, they have their limitations. Ramanunny et al. (2021) reported a range of shortcomings, including associated noxiousness, trouble with scaling up, catering to silver and gold nanoparticles in the size range between 1–100 nm. In addition to these, copper nanoparticles are also implicated in the treatment of skin diseases (Rajeshkumar et al., 2019). Efficacy of gold and silver nanoparticles in wound healing is shown in Figures 1G,H,K–M.

### Metal Oxide Nanoparticles

Among metal oxide nanoparticles commissioned in the therapy of dermatological issues, as well as promoting wound healing, oxides of metals such as zinc, iron, aluminum, magnesium, copper, among others, have found applications [(Salatin et al., 2019; Naskar and Kim, 2020); and references therein]. However, in this context, particular focus has been laid on zinc and iron oxide nanoparticles (Mihai et al., 2019; Naskar and Kim, 2020). Applicability of zinc oxide nanoparticles has been boosted based on the ability to fabricate innovative wound dressings, bi-metal synthesis of “core-shell nanocomposites”, triggering punctures in bacterial cellular membranes, production of hydrogen peroxide, high absorbing capacity of wound exudates, promoting blood clots, among others (Mihai et al., 2019; Salatin et al., 2019). Salatin et al. (2019) further reports that although these metallic oxide nanoparticles show better antibacterial features against Gram positive bacteria (*S. aureus, Pseudomonas aeruginosa*), they do suffer from challenges, including frequent problems of agglomeration. Additionally, there are associated toxicity issues, as reviewed by Naskar and Kim (2020), who also reported that a multicomponent gel with incorporated zinc oxide nanoparticles, chitosan and an antibiotic—gentamicin, demonstrated interactive actions against bacteria, thereby stimulating wound healing. Nevertheless, the lethal effects of metal and metal oxide nanoparticles should not be overlooked in the context of their regular exploitation, which may manifest in diverse, but abominable outcomes for the host species, including cell death, DNA and cellular membrane damage, oxidative stress generated by reactive oxygen species, amongst others. It is not straightforward to counter cytotoxicity, considering the wide range of influencing factors, such as size, shape, concentration, target or host organism (species), type of nanoparticle, mode of delivery, mode of synthesis, just to name a few. This is also shown in Table 1, which displays the various inorganic nanoparticles discussed in this minireview, along with their representative characteristics. For instance, in plants, silver, copper/copper oxide and zinc oxide nanoparticles demonstrate toxicity effects at threshold concentrations of 0.03 mg/ml, 0.2 mg/ml and 2000 ppm, respectively (Rastogi et al., 2017). In this regard, up to date and insightful discussions regarding the inorganic nanoparticle dependent risk profiles, including concentration influenced toxicity, can be found in recent reviews (Wang et al., 2018; Sengul and Asmatulu, 2020). Contextually, the aspect of neonatal toxicity, attributing to the administration of metal and metal oxide nanoparticles, has also received attention in animal models (Brohi et al., 2017). In one very recent study, Becaro et al. (2021) reported the administration of silver nanoparticles (size: 2–20 nm; dose: 0, 0.001, 0.003 and 0.005 mg/kg/day) in pregnant rats, and their influence on the sexual maturity of the neonatal. Even at the highest concentration, although there were no serious toxicity concerns, which could be an early guideline for human testing, a delay in sexual maturity was reported. A while ago, Alsalhi et al. (2016) reported the influence of administration of biosynthesized, “green” silver nanoparticles (using *Pimpinella anisum* seed extract; average diameter: 3.2–16 nm) on human neonatal *in vitro* toxicity. The authors tested several diverse nanoparticle concentrations and concluded that heightened neonatal cytotoxicity occurs at doses exceeding 0.01 mg. At lower doses, only limited antagonistic effects on cellular proliferation could be noted.

**TABLE 1:** Representative characteristics of a few metal and metal oxide nanoparticles, administered either in isolation, or as composites/conjugates, including the targeted hosts and proposed or demonstrated dermato-cosmetic applications. Abbreviation: NPs—Nanoparticles.

Inorganic NPs	Type	Intended applications	Size/size range in diameter	Host	Min. Dose/Dose/Concentration used without toxicity	Remarks	Reference
Gold NPs	Metal	Wound healing, antibacterial properties	28.9–37.65 nm	Mice	0.0005 mg	Administered via thermosresponsive gels	Arafa et al. (2018)
Gold NPs	Metal	Topical psoriasis treatment	4–5 nm	C57BL/6 wild-type mice	No reported toxicity or concentration-toxicity association	For delivery of Methotrexate drug	Besser et al. (2016)
Gold NPs	Metal	Gene regulation/topical psoriasis treatment	10–15 nm	IMQ mice model of psoriasis	50 nM; No reported toxicity	For delivery of siRNA	Nemati et al. (2017)
Silver NPs	Metal	Wound healing	20–40 nm	Sprague-Dawley (SD) rats	0–20 ppm; toxicity not specified	Administered *via* collagen/chitosan scaffolds	You et al. (2017)
Silver NPs	Metal	Numerous including dermato-cosmetic	10–80 nm	Bacteria, yeast, algae, crustaceans, mammals	0.1 mg Ag/L (crustaceans, algae) −26 mg Ag/L (mammalian cells)	Size dependent toxicity analysis (<10 nm perceived non-toxic)	Ivask et al. (2014)
Silver NPs	Metal	Anti-inflammatory; treatment of psoriasis lesions	20–80 nm	Rats (paw tissues), humans	No cytotoxicity at < 0.0237 mg/ml concentration	Green synthesis: using natural berry extract rich in antioxidants	David et al. (2014)
Gold NPs Silver NPs	Metal	Anti-inflammatory; treatment of psoriasis plaques	13–52 nm (Au-NPs) 9–82 nm (Ag-NPs)	Humans	0.165 mg/ml (Au-NPs); 0.19 mg/ml (Ag-NPs). No noteworthy cyctotoxicity	Green synthesis: using polyphenol rich extract (*Cornus mas*)	Crisan et al. (2018)
Copper NPs	Metal	Antibacterial and antioxidant properties, numerous biomedical applications	60–90 nm	Not applicable (only *in vitro* tests)	0.02–0.1 mg/ml, toxicity not specified	Green synthesis: using Cissus arnotiana medicinal plant extract	Rajeshkumar et al. (2019)
Zinc oxide NPs Titanium dioxide NPs	Metal oxide	Cosmetics, dermato-cosmetic	≈30 nm (ZnO NPs) ≈50 nm (TiO_2_ NPs)	Wistar albino rats (male)	Concentration- toxicity association not specified	Administered *via* sunscreen cream	Singh and Nanda (2014)
Iron oxide NPs	Metal oxide	Topical	11 nm (solid state); ≈78 nm (hydrodynamic)	Not applicable (only *in vitro* tests)	Concentration-toxicity association not specified	Administered *via* Pluronic F127 hydrogel	Gonçalves et al. (2017)
Silicon dioxide NPs	Metal oxide	Cosmetics, dermato-cosmetic	≈10 nm	Human (sensory analysis)	4%; toxicity not specified	Administered *via* Pickering emulsions	Terescenco et al. (2020)

## Cosmetic Interventions

### Metal Nanoparticles

Applications of inorganic nanoparticles in the cosmetic industry encompass diverse domains, including lip, nail, hair and skin care, and have seen an upsurge in the last few decades. This is predominantly attributing to the emergence of highly efficient ingredients and carrier vehicles for the targeted delivery of active ingredients, including nanoemulsions, nanoliposomes, nanocapsules, niosomes, among others, which enable these to be perceived as cosmeceuticals. According to the authors, in terms of pure metallic nanoparticles and in resemblance with the dermatological applications, silver and gold nanoparticles have garnered maximum attention as novel cosmeceuticals, finding applications as anti-perspiration sprays and age-delaying creams. Fytianos et al. (2020) reported that silver nanoparticles have been reported to demonstrate stabilizing and antimicrobial actions in cosmetic applications. However, the authors also note that gold and silver nanoparticles show a fundamental dissimilarity with regards to their incorporation in cosmetic products in terms of aggregation. Specifically, gold nanoparticles do not tend to aggregate due to their higher electro kinetic surface potential. Kaul et al. (2018) have collated a list of advertised gold nanoparticle containing cosmetic formulations with a wide variety of personal skin care to therapeutic interventions, which the authors attribute to a plethora of factors, including less toxicity, high robustness (gold-sulphur linkages), chemical unresponsiveness, among others. However, the strongest support in favor of these inorganic nanoparticles are their wide-ranging actions against fungi and bacteria, recuperating blood circulation, ability to counter inflammation and sepsis (Kaul et al., 2018; Niska et al., 2018). Additionally, the small size of the gold nanoparticles facilitating penetration and greater drug stuffing competence, are welcome features as cosmeceuticals (Kaul et al., 2018).

### Metal Oxide Nanoparticles

The applications of titanium dioxide and zinc oxide nanoparticles are highlighted in skin care products, for instance by making the sunscreen items less oily and less malodourous (Kaul et al., 2018). The same authors commented on the usefulness of a combination of silver and zinc/other metal oxide nanoparticles in nail care applications, by conferring antifungal features. From another perspective, the protective characteristics of the zinc and titanium dioxide nanoparticles against several strains of *Staphylococcus* and *Streptococcus* have been identified (Niska et al., 2018). While definitive conclusions regarding the exact antimicrobial activities or their mechanisms are yet to be established, the authors collated reports pointing toward several probable directions. One amongst them is bacterial cell inactivation via amalgamation of nanometal and thiol groups on the cell wall peptidoglycan (Ivask et al., 2014). Perennially favourite metal oxide nanomaterials, for instance zinc and iron oxide nanoparticles, have also been associated with inhibitory actions against the development of bacterial biofilms, as potent cosmeceutical agents (Niska et al., 2018). Efficacy of metal oxide nanoparticles in a topical cosmetic application is shown in Figures 1I,J. The key dermato-cosmetic interventions discussed in this minireview are schematically represented in Figure 1A.

## Significance of Rheological Assessments

The metal and metal oxide nanoparticles, frequently utilized in therapeutic applications against various skin disorders via a wide range of drug delivery vehicles and scaffolds, need to be rigorously characterized to get an understanding of their phase transitions, processability under different operating environments, mechanical properties under stress, as well as temporal and temperature fluctuations. In this context, we will focus only on the rheological characterizations, which not only reveal real material properties under small to large scale deformations, but additionally throw light on microstructure-bulk mechanical property relationship, thermal and deformation stability, yielding under high stress, among others (Goudoulas et al., 2018; Pan et al., 2018; Pan et al., 2019b; Dakhil et al., 2021). For instance, Wong et al. (2016) investigated the rheology of nanocomposites and nanofluids containing zinc oxide nanoparticles, and reported that efficient dispersal and optimized proportion of the same maximize the absorption of UV radiations. This facilitates the development of sunscreen products as cosmeceuticals. Arfat et al. (2017) reported development of bionanocomposites using copper-silver nanoparticles, and extensive rheological characterizations of the same. The authors note that the incorporation of the bimetallic nanoparticles enhanced their mechanical features, thermal robustness, glass transition temperature, among others. Gonçalves et al. (2017) carried out oscillatory rheological measurements to characterize iron oxide (superparamagnetic) nanoparticles in distinct hydrogels intended toward topical applications. Their measurements demonstrated the efficiency of the technique to display the dependence of the dynamic moduli of the fabricated nanoparticle-based hydrogels on the temperature and intracycle linear viscoelastic response as a function of angular frequency. From a cosmetic perspective, rheometry is an efficient tool to regulate the thickness of the nanoparticle incorporated thin films, with distinct sunscreen applications, as reported very recently (Sapcharoenkun et al., 2020). Rukmanikrishnan et al. (2020) have recently shown shear thinning behavior of polysaccharide-based nanocomposites containing zinc oxide nanoparticles, with a direct dependence of the magnitude of the viscoelastic moduli on the proportion of zinc oxide. The authors additionally note that such nanocomposites offer better UV protective and water and heat resisting features, compared to the ones with the nanoparticles. Terescenco et al. (2020) reported very recently the synthesis of cosmetic pickering emulsions containing three metal oxides (titatnium, zinc and silicon) and their characterization via a few high throughput techniques, including rheology. The authors noted that the sensory features of the modified emulsions are directly governed by the material properties of the particles and can be tuned to suit anticipated applications. Interestingly, integration of aluminum oxide nanoparticles into surfactants have been shown to enhance their viscosity, without a noteworthy influence on their viscoelasticity (García and Saraji, 2020). Finally, of late, more and more of the dermatocosmetic applications involving metal or metal oxide nanoparticles, are increasing focusing on “green” fabrication technique to facilitate sustainability. Rheology has also shown to be beneficial for their post-synthesis assessments, to safeguard applications, as shown by Nakhjavani et al. (2017) via the manufacturing and evaluation of antibacterial potential of the silver nanoparticles from the leaves of green tea. It may be noted that in addition to the rheological assessments, several other high-end techniques, including Fourier Transform Infrared (FTIR) spectroscopy, Electron and Atomic Force Microscopy, thermal (Differential Scanning Calorimetry) and photothermal characterizations, thermogravimetric analysis, *in vitro* drug penetration or release tests, amongst others, are in routine use to characterize the inorganic nanoparticle conjugates, composites, alloys, and complexes. These characterizations and their rational interpretations facilitate their process optimization and target utilization in a wide range of dermato-cosmetic applications. For instance, optical properties such as shape and size of metal and metal oxide nanoparticles, have been reported to intensely regulate their mechanical properties, skin penetration attributes, and numerous other biomedical applications (Tak et al., 2015; Hossain et al., 2019). Additionally, conjugates of metal (silver, gold, palladium) and metal oxide (molybdenum oxide, tungsten oxide, iron oxide) nanoparticles have been positively implicated towards photothermal treatments (Wang et al., 2021), including dermatological complications such as skin cancer (Kim et al., 2021). A few representative rheological characterizations of metal oxide nanoparticles are shown in Figures 1C,D,F.

## Psoriasis: A Case Study

Psoriasis is a chronic, auto-immune inflammation disorder of the skin, predominantly revealing as red (erythematous) or flaking (scaly) lacerations that although could be confined to any human body part, generate more frequent manifestations at the joints and scalp (Pradhan et al., 2018; Fereig et al., 2020). Although numerous factors, including smoking, anxiety, alcohol intake, genetic background, amongst others, can be attributed to its trigger, the exact cause has not yet been established (Fereig et al., 2020). However, the authors report that mainly three aspects contribute to its fundamental pathogenesis: pro-inflammatory cytokine synthesis, angiogenesis, and keratinocyte hyperproliferation. The authors also note that at the upper bracket, it currently affects nearly ∼10% of the global population, of which, ∼5% caters exclusively to the developing nations. The extensive traditional rehabilitation (topical, photo- and systemic therapies) targeted towards the management of psoriasis, is grossly influenced by its pathophysiology, and is challenged by both curative (poor drug penetration due to skin hardening, high costs) as well as psychosomatic (poor life quality due to long-lasting reappearance, and subsequent melancholy) factors (Pradhan et al., 2018). In this regard, the upsurge in state-of-the-art, innovative diagnostic and therapeutic strategies to tackle the ailment, implicates extensive nanodrug administration, including metallic nanoformulations (Pradhan et al., 2018; Fereig et al., 2020). This makes psoriasis a typical case study to study the influence of nanoparticle-based therapy on skin disorders. Bessar et al. (2016) reported on the development of gold nanoparticles with methotrexate for the management of psoriasis. The authors noted the higher targeted delivery potential of methotrexate in combination with the gold nanoparticles compared to formulations devoid of them, as well as their non-toxic nature. David et al. (2014) reported on the fabrication of silver nanoparticles incorporated with European blackberry fruit isolate for treating psoriasis. The authors noted the noteworthy inflammation countering potential of the nanoparticles, as well as the diminished swelling, coupled with alleviated quantities of cytokines in the tissues. Contextually, Crisan et al. (2018) have also demonstrated the efficacy of polyphenol rich berry isolates loaded onto silver and gold nanoparticles to lower the extent of inflammation in the treatment of psoriasis. Further, Nemati et al. (2017) amalgamated two diverse strategies, i.e., gene therapy and drug delivery *via* gold nanoparticles to treat “plaque psoriasis” using short interfering RNA or siRNA applications, and in the process, absconded the troubles of encountering cell barricades as well as nuclease digestion. The authors reported that the synthesized gold nanoparticles proved to be non-toxic with a size range of 10–15 nm, with a noteworthy reduction of gene expression. Perde-Schrepler et al. (2016) fabricated gold nanoparticles containing corneal cherry isolates (demonstrated free radical scavenging, inflammation countering and anticarcinogenic activities) *via* a “green” approach towards the treatment of skin cells. The authors note that with additional testing and optimization, these are potential candidates for effective dermatological malfunction treatments. The efficacy of metal nanoparticles in the treatment of psoriasis is shown in Figure 1E.

## Outlook and Forthcoming Prospects

We note that despite significant, recent advancements in the utilization of metal and metal oxide nanoparticles in diverse topical therapeutic applications, challenges persist. For instance, much needs to be achieved regarding the precise mode of action of metal oxide nanoparticles in the context of reactive oxygen species synthesis and associated therapy protocols, with a focus on area-defined management and toxicity assuagement. The premise of *in vitro* investigations and “single-target” bacteria, concerning wound recovery applications, should be extended to both skin microbiota in general, and gram negative and positive bacteria in specific, with a greater focus on *in vivo* studies. In the area of cosmeceuticals, applications of innovative “nanosized metal pigments” should be extensively investigated in addition to zinc oxide and titanium dioxide nanoparticles. Moreover, the unfavorable response toward the utilization of real skin samples for drug penetration and release studies could be improved via employability of *in vitro* models or scaffolds made of natural polysaccharides. Particularly on this front, careful research endeavors are warranted to realize the full potential of multifunctional nanomaterials, novel composites, inorganic nanocomplexes, alloys, amongst others, towards dermatocosmetic applications. For instance, as discussed earlier, preliminary studies have indicated higher drug delivery efficiency, as well as sustained release of the active drug ingredients, when certain metal or metal oxide nanoparticles are conjugated with natural biopolymers, in order to fabricate novel polymer-nanoparticle composites. Additionally, the positive implications of certain alloys (such as titanium) towards advanced skin applications (as implant biomaterials) have not been overlooked. Nevertheless, in this regard, the mechanical attributes of the novel continuous phase matrices need to be thoroughly examined and understood, and compared with pre-established thresholds. The issue of toxicity remains perhaps the most critical aspect to scrutinize, with viewpoints from the mode of synthesis of inorganic nanomaterials, *in vivo* vs *in vitro* toxicity, influence of size, shape and processing conditions on the regulation of toxicity, among others. This especially holds true for the multifunctional nanomaterials and alloys, where size, shape and concentration of the constituent nanoparticles heavily influence the cytotoxicity outcomes. As is perceived, the prominence of the concern over nanoparticle toxicity, above everything, is its ability to impact human health in profoundly adverse manners. It is thus critical that more stringent and regulated *in vivo* and *in vitro* studies are conceptualized and carried out, especially involving human hosts, and human/mammalian cell lines, in order to adequately evaluate and mitigate the toxicity issues. Other than psoriasis, the inorganic nanoparticle laden novel drug carriers will demonstrate noteworthy applications in the treatment of skin carcinogenesis. Tailored anti-cancer therapy will be heavily influenced by drug pharmacokinetics and pharmacodynamics, selection of particular biomarkers, as well as tunable functional characteristics of the nanoparticle mediated drug agents. Elucidation of the molecule-level machinery of the nanomaterial activity on human physiology, is critical for their maximized utilization in skin and cosmetic applications. Once carefully adjusted at the lab scale, attention must also be directed towards scaling up and commercialization of such novel nanocarriers, which currently presents a significant void in fulfillment. Additionally, implementation of quality by design and process analytical technology aspects into the regulation of the scaling up protocols should not be overlooked, to achieve process and product optimization.
